# Depression and cause-specific mortality in an ethnically diverse cohort from the UK: 8-year prospective study

**DOI:** 10.1017/S0033291718002210

**Published:** 2018-09-05

**Authors:** Jayati Das-Munshi, Chin-Kuo Chang, Peter Schofield, Robert Stewart, Martin J. Prince

**Affiliations:** 1 Department of Health Services and Population Research, King's College London, Institute of Psychiatry, Psychology & Neuroscience, London, UK; 2 South London and Maudsley NHS Foundation Trust, London, UK; 3 Department of Psychological Medicine, King's College London, Institute of Psychiatry, Psychology & Neuroscience, London, UK; 4 Department of Health and Welfare, University of Taipei, Taipei City, Taiwan; 5 King's College London, Primary Care and Public Health Sciences, London, UK

**Keywords:** All-cause mortality, depression, ethnicity, natural cause mortality, unipolar depression, unnatural cause mortality

## Abstract

**Background:**

Depression is associated with increased mortality, however, little is known about its variation by ethnicity.

**Methods:**

We conducted a cohort study of individuals with ICD-10 unipolar depression from secondary mental healthcare, from an ethnically diverse location in southeast London, followed for 8 years (2007–2014) linked to death certificates. Age- and sex- standardised mortality ratios (SMRs), with the population of England and Wales as a standard population were derived. Hazard ratios (HRs) for mortality were derived through multivariable regression procedures.

**Results:**

Data from 20 320 individuals contributing 91 635 person-years at risk with 2366 deaths were used for analyses. SMR for all-cause mortality in depression was 2.55(95% CI 2.45–2.65), with similar trends by ethnicity. Within the cohort with unipolar depression, adjusted HR (aHRs) for all-cause mortality in ethnic minority groups relative to the White British group were 0.62(95% CI 0.53–0.74) (Black Caribbean), 0.53(95% CI 0.39–0.72) (Black African) and 0.69(95% CI 0.52–0.90) (South Asian). Male sex and alcohol/substance misuse were associated with an increased all-cause mortality risk [aHR:1.94 (95% CI 1.68–2.24) and aHR:1.18 (95% CI 1.01–1.37) respectively], whereas comorbid anxiety was associated with a decreased risk [aHR: 0.72(95% CI 0.58–0.89)]. Similar associations were noted for natural-cause mortality. Alcohol/substance misuse and male sex were associated with a near-doubling in unnatural-cause mortality risk, whereas Black Caribbean individuals with depression had a reduced unnatural-cause mortality risk, relative to White British people with depression.

**Conclusions:**

Although individuals with depression experience an increased mortality risk, marked heterogeneity exists by ethnicity. Research and practice should focus on addressing tractable causes underlying increased mortality in depression.

## Background

Depression has been described as a ‘life threatening disorder’ (Cuijpers and Smit, 2002), with a population attributable risk for mortality comparable with diabetes mellitus (Pratt *et al*., 2016). Excess risks of mortality have been described in community studies for major depression (Saz and Dewey, 2001; Cuijpers and Smit, 2002; Cuijpers *et al*., 2014), subclinical depression (Cuijpers and Smit, 2002) and depressive symptoms (Russ *et al*., 2012). Although deaths from unnatural causes and suicide are elevated in depressed populations relative to non-depressed reference populations (Harris and Barraclough, 1998), a large proportion of deaths are accounted for through common preventable physical conditions such as cardiovascular disease (Wulsin *et al*., 1999), with an elevated mortality risk still evident four decades after the initial depressive episode (Wyman *et al*., 2012).

Few studies have assessed the association of depression with mortality in ethnically diverse populations. Authors of a previous systematic review indicated heterogeneity in the mortality risk associated with depression across populations, leading to speculation on the role of ‘cultural factors’ in mortality risk (Cuijpers and Smit, 2002). Most studies that have assessed mortality in ethnically diverse populations have focused on suicide mortality, largely within US populations (Oquendo *et al*., 2001; Zivin *et al*., 2007). As physical conditions such as cardiovascular disease and diabetes mellitus are more prevalent among certain ethnic minority groups in the USA, Europe and elsewhere (Yusuf *et al*., 2001), understanding the interplay of these with depression and mortality risk remains an important issue needing further understanding.

Populations have become increasingly ethnically diverse within the last two decades, yet most studies of mortality in depression have not been able to reflect this, raising concerns over generalisability with implications for clinical practice and public health policy. A better understanding of the differences in mortality in depression among ethnically diverse populations may also serve to highlight potentially modifiable risks for populations of any ethnicity and could help to inform the development of interventions.

To address this gap in knowledge, we undertook analyses of a cohort of individuals with a diagnosis of unipolar depression, using electronic health records from a large secondary care mental healthcare provider, which provides near-monopoly mental health coverage to a catchment area of 1.34 million people in south-east London, an ethnically diverse inner city area in the UK (Perera *et al*., 2016). This study is part of a larger programme of work, designed to assess ethnic minority physical health inequalities in mental illness (Das-Munshi *et al*., 2016).

We sought to establish:

(1) All-cause and cause-specific mortality by ethnicity in people with depression under secondary mental healthcare.
(2) Ethnicity and other social, clinical and demographic risk factors for all-cause and cause-specific mortality in people with unipolar depression, taking into account potential confounders.

## Methods

### Setting and participants

The South London & Maudsley NHS Foundation Trust (SLaM) is a large mental healthcare provider providing near-monopoly coverage for around 1.34 million residents of a defined geographic catchment in southeast London (Perera *et al*., 2016). From 2006, SLaM has used a fully electronic health records system with a separate interface for research, known as the Clinical Records Interactive Search system (CRIS). Researchers using CRIS are able to specify search terms to extract information from structured fields. In addition, a range of natural language processing (NLP) algorithms has been developed to extract information from the free text of clinical records (Perera *et al*., 2016). Individuals had to be aged 15 or over at the time of diagnosis.

### Major depression and other clinical diagnoses

NLP algorithms developed in CRIS include an application for ascertaining text describing assigned diagnoses which have been commonly used in analyses to supplement diagnostic information recorded in structured fields within the electronic health record (Perera *et al*., 2016). For this analysis, using this algorithm in combination with structured fields, we searched for individuals with a primary clinical diagnosis of depression according to the *International Classification of Disorders-*10 (ICD-10) (World Health Organisation, 1993) (codes F32-F33), with index date of diagnosis, which led to the creation of a longitudinal cohort who were followed to 12 April 2015. We searched diagnoses fields and excluded individuals with any of the following comorbid diagnoses at the same time point or if occurring after the index depression diagnosis: dementia, learning disabilities, eating disorders, severe mental illnesses (including schizophrenia-spectrum and bipolar disorders) and Attention Deficit Hyperactivity Disorders from the cohort. We also used the secondary diagnoses fields to ascertain individuals with comorbid anxiety (ICD-10 codes: F40-F48 ‘Neurotic, stress-related and somatoform disorders’ including anxiety disorders). The presence of comorbid alcohol and substance use (ICD-10 codes F10-F19) at any point was noted and used to further inform analyses.

A validation check was conducted on diagnoses derived from NLP for this analysis, selecting a random sub-set of 100 records which were assessed against a ‘gold standard’ of ICD-10 depression diagnoses, ascribed by a senior clinician (consultant psychiatrist-JD), who manually reviewed clinical records for diagnoses. Records in the full sample were rated blind to other sociodemographic characteristics (age, gender and ethnicity). The sensitivity of clinical diagnoses derived through NLP algorithms was 89.6% (95% CI 77.3–96.5%), the specificity was 88.0% (95% CI 75.6–95.5%), and the positive predictive value was 87.8% (95% CI 75.2–95.4%). Performance of the NLP algorithm for diagnosis was broadly similar across each of the ethnic minority groups (online Supplementary Table S4).

### Exposures and covariates

Details on age, gender and marital status (married, divorced/separated, single, widowed and civil partnerships) were derived from structured fields. Self-ascribed ethnicity, based on the Office for National Statistics 2001 ethnicity categories (The Office for National Statistics, 2015) were derived from structured fields and grouped into the following: White British, Irish, Black Caribbean, Black African and Chinese. People of Indian, Pakistani and Bangladeshi ethnicity were grouped together as South Asian (Bhopal, 2004): a decision taken following assessment of sub-group sample sizes. Addresses of individuals were linked to the neighbourhood Index of Multiple Deprivation (IMD) score at the Lower Layer Super Output Area (LLSOA) (Office for National Statistics, Archived on, 6 Jan, 2016,). The IMD assesses disadvantage across multiple domains (income, employment, education, housing, crime, living environment and health deprivation) from national Census data (Noble *et al*., 2006), and the LLSOA is a statutory geographical area which has between 400 and 1200 households (Office for National Statistics, Archived on, 6 Jan, 2016,).

### Cohort follow-up and mortality

Date of first recorded depression diagnosis in SLaM denoted the date which individuals came into the study. Individuals were followed until the end of the study (31 December 2014), death or emigration, whichever came first. A separate file detailing date and cause of death, as well as emigrations out of the UK, was provided by the Office of National Statistics (ONS) and linked to CRIS, using National Health Service (NHS) identification numbers. A separate code for ‘cancelled ciphers’ was also provided, which indicated deregistration. These codes are a reasonable proxy for emigrations out of the UK (National statistics, 2007). ICD-10 codes were used to determine the cause of death which were grouped into all-cause (codes A00-R99; U00-Y89), natural cause (A00-Q99) and unnatural/external causes (including deaths from suicide and from accidents and assaults) (U509, V01-Y89) mortality and deaths not elsewhere classified (R00-R99). Cause-specific codes were further grouped as deaths from cancers (C00-D48), respiratory disorders (J00-J99), circulatory disorders (I00-I99), diabetes mellitus (E10-E14) and suicide (X60-X84, Y10-Y34).

### Statistical analyses

Indirect standardisation by age and gender for mortality was used to derive standardised mortality ratios (SMRs) with 95% confidence intervals (95% CIs). SMRs were calculated based on the counterparts (deaths and resident population) in England and Wales, using the mid-point of the follow-up period in 2011. This process of standardisation leads to an estimation of mortality ratios (observed deaths in the study population divided by expected deaths in the reference population) which takes into account the confounding effects of age and gender. To derive SMRs, age was determined as the mid-point of the observation period or date of diagnosis of mental disorder, if diagnosis occurred after the mid-point. Age was broken down into 10-year bands, corresponding to the reference population age-groups. Because mortality of the standard population was given for 1 year but our target population was observed for up to 8 years, weights to account for the length of follow-up period were derived by taking the mean observation period contributed by individuals within each corresponding age- and gender-band in the cohort. Each weight was multiplied by the number of deaths recorded in each corresponding band for the standard population, providing an estimation of the expected number of deaths in the observation period.

Cox regression was used to assess the association of exposures with mortality (all-cause, natural-cause and unnatural-cause) over time. Proportional hazards assumptions were checked by assessing interactions with survival time, examining Schoenfeld residual plots and by testing for a zero-slope in scaled residuals. Lexis expansion was used to derive the time-changing variables of age and time since diagnosis (in tertiles: 0–1, 1–3, 3+ years). The expansion of the age and ‘time since diagnosis’ variables, through the process of Lexis expansion, permitted an assessment of an interaction with time-changing variables in the models, with rates assumed to be constant within time bands. Where proportional hazards assumptions were violated, an interaction with time was fitted. The association of age with mortality as a continuous linear term and as a quadratic term with mortality was assessed, using likelihood ratio tests (LRTs). In final models, age was included as a quadratic term as LRTs suggested an improved fit over age as a linear term.

To assess associations with natural-cause and unnatural-cause mortality outcomes, a modified Cox regression taking into account competing risks, was used (Fine and Gray, 1999). These models take into account the likelihood of the competing event occurring (events which remove study participants from ‘being at risk’ of the event of interest; e.g. through death from other another cause) (Fine and Gray, 1999). We first assessed the association of independent variables with natural-cause mortality, competing with unnatural-cause mortality risks. We next assessed the association of independent variables with unnatural-cause mortality, competing with natural-cause mortality risk. Sub-hazard ratios (HRs) with 95% confidence intervals based on robust standard error estimations were generated. Wald tests were used for hypothesis testing. Analyses were conducted in Stata/SE 13.1.

### Sensitivity analyses

The first generation ethnic minorities may be more likely to migrate back to their country of origin when unwell and/or prior to death, which may lead to a biased under-estimation of mortality risk, through a ‘numerator-denominator mismatch’ (Razum, 2006). Therefore, as a sensitivity analysis, we re-assessed associations for ethnicity and all-cause mortality by using competing-risks regression, specifying emigration out of the cohort as a competing event as opposed to as a censored event.

In further sensitivity analyses, we assessed differences in standardised all-cause mortality ratios when age- and sex- standardised to the local area, and compared these to estimates standardised to the population of England and Wales.

## Results

**Fig. 1.**
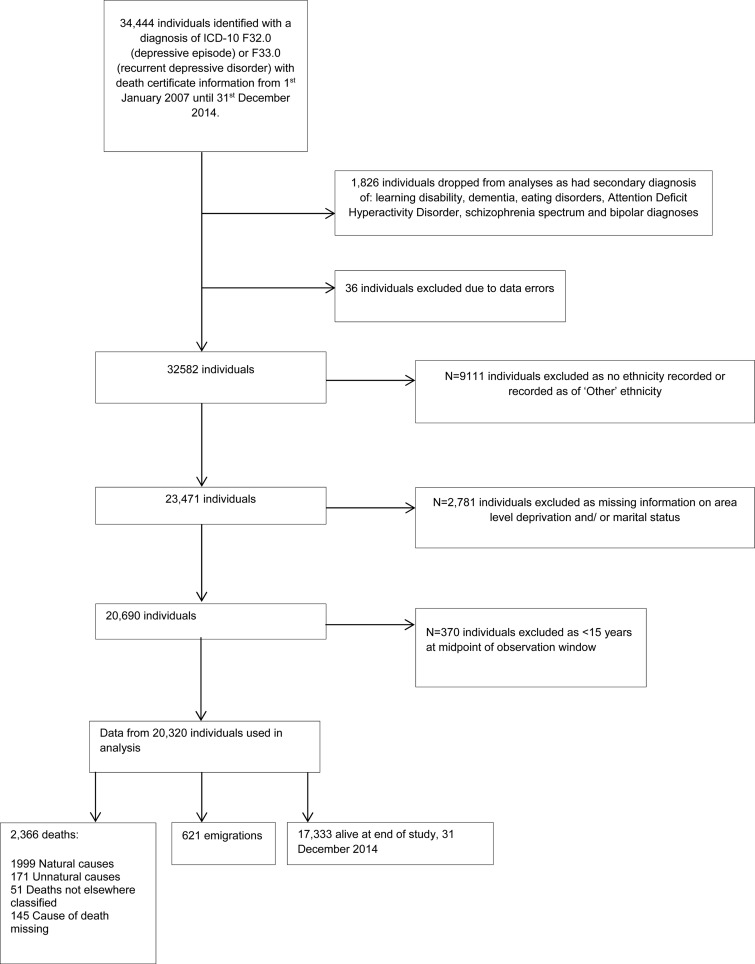
Flowchart of participants.

In total, there were data from 20 320 individuals with depressive diagnoses, contributing 91 635 person-years at risk for mortality, who were followed to the end of the study on 31 December 2014, or who died or emigrated before the study end date (Fig. 1). Mean follow up time was 4.5 years. Table 1 summarises the demographic features of the sample. There were 2366 deaths (11.6%) from all causes, which included 1999 deaths from natural causes (9.9%) and 171 deaths from unnatural causes, including suicide (0.9%), over the 8-year follow-up period. Just over two-thirds of the sample were White British, more than half the sample were of single marital status, 13% had a current or previous alcohol/substance misuse disorder.

**Table 1.** Demographic features of the cohort with unipolar depression

	*N*	%
Total sample	20 320	100.0
Age in years [median (IQR)]	43.6	(40.0, 57.7)
Ethnicity		
White British	13 903	68.4
Irish	754	3.7
Black Caribbean	2942	14.5
Black African	1863	9.2
South Asian	741	3.6
Chinese	117	0.6
Sex		
Female	12 658	62.3
Male	7662	37.7
Marital status		
Married/cohabiting	2880	14.2
Divorced/separated	2547	12.5
Single	11 136	54.8
Widowed	1745	8.6
Civil partnership	2012	9.9
Area-level deprivation^a^		
5: Most deprived fifth	4019	19.8
4: Fourth	4010	19.7
3: Third	3991	19.6
2: Second	4054	20.0
1: Least deprived fifth	4246	20.9
Comorbid anxiety		
None	19 080	93.9
Comorbid anxiety	1240	6.1
Comorbid alcohol/substance misuse		
None	17 737	87.3
Alcohol/substance misuse	2583	12.7
Deaths		
All-cause	2366	11.6
Natural causes	1999	9.8
Unnatural causes	171	0.8
Deaths not elsewhere classified	51	0.3

a Assessed by the index of multiple deprivation in fifths.

### Cause-specific mortality standardised to the population of England and Wales

Compared with the general population of England and Wales in 2011, age- and sex- SMRs were elevated for most causes of death across the sample (Table 2). SMRs for all-cause mortality were increased more than two-fold in the full sample and across each of the ethnic minority groups. Unnatural cause mortality was elevated more than five-fold in the full sample and suicide mortality was elevated more than ten-fold in the full sample. Deaths from natural causes varied from 1.84 (full sample, cancers), to over two-fold (from cardiovascular causes, including stroke and myocardial infarctions) and over three-fold (diabetes mellitus), across the full sample.

**Table 2.** Cause-specific standardised mortality ratios in unipolar depression, by ethnicity (*N* = 20 320)

Totals (N)	Full sample	White British	Black Caribbean	Black African	South Asian	Irish	Chinese
	20 320	13 903	2942	1863	741	754	117
ALL-CAUSE							
Observed/Expected	2366/929	1966/747	153/72	45/21	53/24	144/63	5/2
SMR (95%CI)	**2.55(2.45–2.65)**	**2.63(2.52–2.75)**	**2.13(1.81–2.50)**	**2.19(1.60–2.94)**	**2.17(1.62–2.83)**	**2.28(1.93–2.69)**	**2.11(0.69–4.93)**
NATURAL CAUSES							
Cardiovascular (includes stroke and myocardial infarction)							
Observed/Expected	622/271	502/221	54/20	6/5	15/7	43/19	2/1
SMR (95%CI)	**2.30(2.12–2.48)**	**2.28(2.08–2.48)**	**2.76(2.07–3.60)**	**1.32(0.48–2.86)**	**2.20(1.23–3.63)**	**2.30(1.66–3.10)**	**2.92(0.35–10.56)**
Diabetes Mellitus							
Observed/Expected	36/10	27/8	5/1	2/0	2/0	0	0
SMR (95%CI)	**3.78(2.65–5.24)**	**3.52(2.32–5.12)**	**6.85(2.23–15.99)**	**10.37(1.26–37.47)**	**8.09(0.98–29.23)**	**–**	**–**
Cancers							
Observed/Expected	481/261	396/204	36/23	14/7	4/8	30/18	1/1
SMR (95% CI)	**1.84(1.68–2.01)**	**1.94(1.75–2.14)**	**1.57(1.10–2.18)**	**1.89(1.03–3.17)**	**0.50(0.14–1.28)**	**1.67(1.12–2.38)**	**1.53(0.04–8.55)**
Respiratory							
Observed/Expected	429/131	377/107	8/9	3/2	11/3	30/9	0
SMR (95% CI)	**3.27(2.97–3.59)**	**3.51(3.17–3.88)**	**0.85(0.37–1.68)**	**1.56(0.32–4.57)**	**3.35(1.67–5.99)**	**3.34(2.25–4.77)**	**–**
UNNATURAL CAUSES							
Observed/Expected	171/33	137/25	7/4	9/2	7/1	10/2	1/0
SMR (95% CI)	**5.09(4.35–5.91)**	**5.46(4.58–6.45)**	**2.00(0.80–4.12)**	**4.31(1.97–8.17)**	**7.00(2.81–14.43)**	**5.61(2.69–10.31)**	**8.41(0.21–46.88)**
Suicide							
Observed/Expected	98/9	77/6	4/1	8/1	2/0	6/0	1/0
SMR (95% CI)	**10.58(8.59–12.89)**	**11.91(9.40–14.88)**	**3.41(0.93–8.74)**	**9.18(3.96–18.08)**	**6.39(0.77–23.06)**	**14.84(5.45–32.31)**	**27.23(0.69–151.7)**
Accidents/assaults							
Observed/Expected	73/24	60/19	3/2	1/1	5/1	4/1	0
SMR (95% CI)	**3.00(2.35–3.77)**	**3.22(2.46–4.14)**	**1.29(0.27–3.77)**	**0.82(0.02–4.57)**	**7.28(2.36–16.99)**	**2.90(0.79–7.43)**	**–**

SMR, standardised mortality ratios- age and sex-standardised to mid-point population of England and Wales; (95% CI) 95% confidence intervals.

Across each of the ethnic minority groups, relative to estimates from the full sample, point estimates for suicide mortality in Irish and Chinese people with depression appeared elevated, although smaller sample sizes limited inferences. Deaths from diabetes mellitus in Black Caribbean [SMR: 6.85 (95% CI 2.23–15.99)], Black African [SMR: 10.37 (95% CI 1.26–37.47)] and South Asian groups with depression [SMR: 8.09 (95% CI 0.98–29.23)] were also markedly elevated. Of note, deaths from respiratory disorders in the Black Caribbean group with depression were lower compared with both the full sample as well as the White British group with depression. Although the precision of estimates was impacted by fewer deaths, the point estimate for SMRs appeared lower for cancer mortality in South Asian people with depression, respiratory mortality in Black Caribbean people with depression and deaths from accidents/other external causes in Black African people with depression (Table 2). Compared with the full sample, point estimates for mortality from unnatural causes (including suicides and accidents/assaults) were lower in Black Caribbean people, as were mortality from cancers in South Asian people with depression (Table 1).

### All-cause, natural and unnatural cause mortality

Table 3 displays the association of main exposures with all-cause mortality. An interaction with ‘time in study’ was fitted for age and gender as proportional hazards assumptions would have otherwise been violated. χ^2^ tests for non-zero slope in Schoenfeld residuals were *p* = 0.285, for the final model, with strong evidence in support of an interaction between sex and ‘time in study’ (*LRT p* *<* *0.001 for sex* × *time interaction,* see online Supplementary Table S1).

**Table 3.** All-cause mortality associations in unipolar depression [2366 deaths out of an overall sample of 20 320 people (11.6%)]

		Deaths (%)	Rate^a^	(95% CI)	Crude Hazard Ratio			Adjusted Hazard Ratio^b^		
					HR	(95% CI)	*p* value	HR	(95% CI)	*p* value
Ethnicity	White British	14.2	31.7	(30.3–33.1)	1.00	REF	.	1.00	REF	.
	Irish	19.2	41.0	(34.8–48.3)	1.28	1.08–1.51	0.0049	0.88	0.74–1.04	0.14
	Black Caribbean	5.2	11.3	(9.7–13.3)	0.36	0.30–0.42	<0.0001	0.62	0.53–0.74	<0.0001
	Black African	2.4	5.1	(3.8–6.8)	0.16	0.12–0.22	<0.0001	0.53	0.39–0.72	<0.0001
	South Asian	7.2	16.2	(12.4–21.2)	0.51	0.39–0.68	<0.0001	0.69	0.52–0.90	0.007
	Chinese	4.3	10.4	(4.3–24.9)	0.33	0.14–0.78	0.012	0.65	0.27–1.57	0.34
Sex	Female	10.5	22.7	(21.5–23.9)	1.00	REF	.	1.00	REF	.
	Male	13.7	31.3	(29.5–33.3)	1.81^c^	1.57–2.08	<0.0001	1.94^c^	1.68–2.24	<0.0001
Relationship status	Married/cohabiting	15.3	28.1	(25.6–30.9)	1.00	REF	.	1.00	REF	.
	Divorced/separated	10.2	20.7	(18.3–23.3)	0.72	0.62–0.85	<0.0001	0.99	0.84–1.15	0.86
	Single	5.5	11.8	(10.9–12.8)	0.41	0.36–0.47	<0.0001	1.17	1.03–1.33	0.019
	Widowed	48.5	127.0	(118.7–135.9)	4.29	3.82–4.81	<0.0001	1.35	1.20–1.53	<0.0001
	Civil partnership	11.0	39.1	(34.2–44.6)	1.26	1.07–1.48	0.0057	1.31	1.11–1.54	0.001
Comorbid anxiety	No comorbid anxiety	12.0	26.5	(25.5–27.6)	1.00	REF	.	1.00	REF	.
	Comorbid anxiety	6.9	15.0	(12.1–18.5)	0.57	0.46–0.71	<0.0001	0.72	0.58–0.89	0.003
Substance misuse	No alcohol/substance misuse	12.3	27.3	(26.1–28.4)	1.00	REF	.	1.00	REF	.
	Alcohol/substance misuse	8.0	16.6	(14.5–19.1)	0.61	0.53–0.70	<0.0001	1.18	1.01–1.37	0.035
Area level deprivation	Most deprived fifth	11.9	25.5	(23.3–27.9)	1.05	1.02–1.08	<0.0001^d^	0.96	0.93–0.99	0.004^d^
		11.0	23.8	(21.7–26.2)						
		10.5	23.1	(21.0–25.5)						
		11.0	24.5	(22.3–26.9)						
	Least deprived fifth	14.0	32.0	(29.6–34.7)						

a Crude rates per 1000 person-years.

b Adjusted for age and all other variables in table, with an interaction between sex and time in study.

c Baseline hazard ratio (at 0–1 years), (see online Supplementary Table S1).

d Linear trend association with mortality, per increase in area-level deprivation (fifths).

*p* values are from Wald tests.

In crude models, except for Irish people with depression in whom the risk was elevated, all other ethnic minority groups appeared to have a reduced risk of mortality relative to the White British group. In the fully adjusted model, this reduced risk persisted in Black Caribbean, Black African and South Asian groups, with similar trends for Irish and Chinese groups. Men with depression were almost twice as likely to die from all causes relative to women with depression, at the start of the study [adjusted HR (aHR): 1.94 (95% CI 1.68–2.24) at 0–1 years], over time this excess risk by sex diminished, reducing to an aHR of 1.34 (95% CI 1.16–1.55), by the end of study in depressed men *v.* women (see online Supplementary Table S1). Relative to people who were married or cohabiting, people who were single, widowed or in civil partnerships appeared to experience an elevated mortality risk over the follow-up period, in adjusted models. The presence of comorbid anxiety disorders was associated with a significant reduction in mortality risk, in adjusted models, whereas the presence of a previous or current history of comorbid alcohol/substance misuse increased the risk of mortality by 18% over the follow-up period. Residence in areas which were less deprived was also associated with a reduced mortality risk in people with depression, relative to those in more deprived areas, in adjusted models (Table 3).

There were similar associations noted with mortality for most of the covariates in analyses of natural cause mortality, taking into account the competing risk of death from unnatural causes (Table 4), with evidence again suggestive of each of the ethnic minority groups within the study with depression (except Irish people with depression) having a reduced sub-HR (sHR) for natural cause mortality in crude and adjusted models, compared to White British people with depression. Associations of relationship status with natural cause mortality in adjusted models differed from the findings for all-cause mortality.

**Table 4.** Natural cause mortality^a^ in unipolar depression [1999 deaths out of an overall sample of 20 320 people (9.8%)]

		Deaths (%)	Rate^b^	(95% CI)	Crude sHR			Adjusted sHR^c^		
					sHR	(95% CI)	*p* value	sHR	(95% CI)	*p* value
Ethnicity	White British	12.0	26.8	(25.6–28.1)	REF			REF		
	Irish	16.7	35.6	(29.9–42.4)	1.32	(1.10–1.57)	0.0003	0.96	(0.82–1.13)	0.60
	Black Caribbean	4.6	9.9	(8.4–11.7)	0.37	(0.31–0.44)	<0.0001	0.68	(0.57–0.80)	<0.0001
	Black African	1.7	3.6	(2.6–5.1)	0.14	(0.10–0.19)	<0.0001	0.47	(0.33–0.66)	<0.0001
	South Asian	5.7	12.8	(9.5–17.3)	0.48	(0.35–0.65)	<0.0001	0.70	(0.53–0.94)	0.016
	Chinese	2.6	6.2	(2.0–19.3)	0.23	(0.07–0.72)	0.011	0.43	(0.14–1.36)	0.15
Sex	Female	8.8	19.1	(18.0–20.3)	REF			REF		
	Male	11.6	26.6	(24.9–28.4)	1.37	(1.26–1.50)	<0.0001	1.48	(1.36–1.61)	<0.0001
Relationship status	Married/cohabiting	13.2	24.2	(21.9–26.8)	REF			REF		
	Divorced/separated	9.0	18.2	(16.0–20.7)	0.74	(0.63–0.87)	<0.0001	0.88	(0.76–1.03)	0.11
	Single	4.3	9.3	(8.5–10.2)	0.38	(0.33–0.43)	<0.0001	0.93	(0.82–1.05)	0.24
	Widowed	41.8	109.4	(101.7–117.7)	4.25	(3.75–4.81)	<0.0001	1.10	(0.98–1.23)	0.11
	Civil partnership	9.5	33.7	(29.2–38.9)	1.23	(1.03–1.47)	0.022	0.79	(0.67–0.93)	0.005
Comorbid anxiety	No comorbid anxiety	10.2	22.4	(21.4–23.4)	REF			REF		
	Comorbid anxiety	5.8	12.7	(10.1–16.0)	0.57	(0.45–0.72)	<0.0001	0.74	(0.59–0.93)	0.008
Substance misuse	No alcohol/substance misuse	10.4	23.2	(22.2–24.3)	REF			REF		
	Alcohol/substance misuse	6.1	12.8	(10.9–14.9)	0.57	(0.49–0.67)	<0.0001	1.12	(0.95–1.31)	0.17
Area level deprivation	Most deprived fifth	9.9	21.3	(19.3–23.5)	1.05	(1.02–1.09)	0.001^d^	0.96	(0.93–0.99)	0.003^d^
		9.5	20.6	(18.6–22.8)						
		8.7	19.3	(17.4–21.5)						
		9.2	20.4	(18.4–22.6)						
	Least deprived fifth	12.0	27.4	(25.1–29.9)						

a Sub-hazard ratios (sHRs) for mortality from natural-causes competing with unnatural-causes.

b Crude rates per 1000 person-years.

c Adjusted for age and all other variables in table.

d Linear trend association with mortality, per increase in area-level deprivation (fifths).

*p* values are from Wald tests.

For unnatural cause mortality (including suicides and deaths from accidents and other external causes), the Black Caribbean group had a reduced risk of death compared with the White British group (Table 5). Relationship status, comorbid anxiety and living in more deprived areas were not associated with an increased risk of unnatural causes mortality in people with depression; however, the presence of comorbid alcohol/substance use and being of male sex were both associated with a near-double increase in the risk of death from unnatural causes.

**Table 5.** Unnatural cause mortality^a^ in unipolar depression [171 deaths out of an overall sample of 20 320 people (0.8%)]

		Deaths (%)	Rate^b^	(95% CI)	Crude sHR			Adjusted sHR^c^		
					sHR	(95% CI)	*p* value	sHR	(95% CI)	*p* value
Ethnicity	White British	1.01	2.2	(1.9–2.6)	REF			REF		
	Irish	1.32	2.8	(1.5–5.3)	1.29	(0.68–2.45)	0.78	1.14	(0.59–2.20)	0.69
	Black Caribbean	0.24	0.5	(0.2–1.1)	0.25	(0.12–0.53)	<0.001	0.30	(0.14–0.66)	0.002
	Black African	0.48	1.0	(0.5–2.0)	0.50	(0.25–0.98)	0.042	0.64	(0.32–1.27)	0.20
	South Asian	0.94	2.1	(1.0–4.5)	1.00	(0.47–2.14)	1.00	1.15	(0.54–2.47)	0.71
	Chinese	0.85	2.1	(0.3–14.7)	0.96	(0.13–6.91)	0.97	1.28	(0.18–9.37)	0.81
Sex	Female	0.58	1.2	(1.0–1.6)	REF			REF		
	Male	1.33	3.0	(2.4–3.6)	2.13	(1.71–3.14)	<0.0001	1.99	(1.45–2.73)	<0.0001
Relationship status	Married/cohabiting	1.11	2.1	(1.5–2.9)	REF			REF		
	Divorced/separated	0.86	1.8	(1.2–2.7)	0.84	(0.49–1.45)	0.54	0.78	(0.45–1.34)	0.36
	Single	0.76	1.6	(1.3–2.0)	0.77	(0.51–1.16)	0.22	0.83	(0.55–1.24)	0.37
	Widowed	1.14	2.9	(1.8–4.5)	1.06	(0.60–1.86)	0.85	0.88	(0.46–1.69)	0.70
	Civil partnership	0.79	2.9	(1.7–4.7)	1.11	(0.60–2.02)	0.74	0.75	(0.41–1.36)	0.34
Comorbid anxiety	No comorbid anxiety	0.87	1.9	(1.6–2.2)	REF			REF		
	Comorbid anxiety	0.73	1.6	(0.8–3.0)	0.87	(0.44–1.70)	0.68	0.83	(0.42–1.62)	0.58
Substance misuse	No alcohol/substance misuse	0.77	1.7	(1.4–2.0)	REF			REF		
	Alcohol/substance misuse	1.47	3.1	(2.2–4.2)	1.92	(1.34–2.75)	*p* < 0.0001	1.77	(1.20–2.62)	0.004
Area level deprivation	Most deprived fifth	0.99	2.1	(1.6–2.9)	1.04	(0.93–1.16)	0.51^d^	1.01	(0.90–1.12)	0.90^d^
		0.57	1.2	(0.8–1.9)						
		0.88	1.7	(1.2–2.5)						
		0.91	2.0	(1.5–2.8)						
	Least deprived fifth	0.94	2.2	(1.6–3.0)						

a Sub-hazard ratios (sHRs) for mortality from unnatural-cause competing with natural-causes mortality.

b Crude rates per 1000 person-years.

c Adjusted for age and all other variables in table.

d Linear trend association with mortality, per increase in are-level deprivation (fifths).

*p* values are from Wald tests.

Sensitivity analyses taking into account emigration out of the cohort as a competing risk for all-cause mortality, had little impact on the estimates displayed in Table 2 (estimates were identical to two decimal places) (online Supplementary Table S2). This confirmed that a higher potential risk of emigration by ethnic minority groups had not significantly biased estimates in these analyses. A further set of sensitivity analyses, whereby all-cause mortality was age- and sex-standardised to the population of the local area, also did not indicate obvious differences in SMRs which were in fact slightly larger (online Supplementary Table S3).

## Discussion

### Main findings

Our findings confirm that in general, irrespective of ethnicity, people with unipolar depression experienced an increased all-cause, natural cause and unnatural-cause mortality risk, relative to the general population. Detailed analyses by cause of death indicated a degree of heterogeneity by ethnicity and in some cases a lower mortality risk in depression for some ethnic groups, compared with the non-depressed reference population. Our longitudinal analyses focusing on individuals with unipolar depression followed over 8 years, indicated a reduced risk of all-cause mortality across ethnic minority groups, relative to the White British group, with depression; this was especially marked for Black African, Black Caribbean and South Asian individuals with depression with similar trends noted for deaths from natural causes. For deaths from unnatural causes, Black Caribbean people with depression had a lower mortality risk relative to White British individuals with depression.

The finding of an increased mortality risk in people with depression is consistent with the literature (Wulsin *et al*., 1999; Saz and Dewey, 2001; Cuijpers *et al*., 2014). Our findings extend these observations and indicate that while this was also the case for the main ethnic minority groups in the study when compared with the general population, within a cohort of individuals with depression there is much heterogeneity by ethnicity in mortality risk.

### Interpretation and relationship to the wider literature

#### Ethnicity and the association with mortality in depression

The finding of a lower mortality risk in ethnic minority groups with depression relative to White British groups with depression, as far as we are aware, has not been reported previously; however, other studies have noted similar associations for severe mental illnesses including the psychoses (Das-Munshi *et al*., 2017*b*) and in the risk of death following self-harm (Turnbull *et al*., 2015). Although the findings in this study may indicate a commonality in mortality experiences related to mental disorders it is possible that the findings may also reflect broader mortality trends in non-depressed migrant populations to the UK (Ikram *et al*., 2016), it was not possible to assess this further by standardising the ethnicity-specific SMRs by the relevant ethnic sub-group, as ethnicity is not routinely recorded on death certificates in the UK (Jarman and Aylin, 2004). Lower mortality risks could be due to ‘healthy migrant’ effects (Ikram *et al*., 2016), however, this possibility seems inconsistent with the observation that healthy migrant/selection effects could operate for mortality outcomes but not for mental disorders and our sample would have also included second-generation ethnic minority groups. This could be explored in future research. Our detailed sensitivity analyses would have ruled out the possibility of migrant groups returning to their country of origin prior to death, leading to a numerator/denominator mismatch (or so-called ‘salmon biases’) (Razum, 2006). It is also possible that ‘risk factors’ for mortality are lower in ethnic minority groups relative to White British people. For example, a study assessing mortality following self-harm episodes found a lower prevalence of risk factors such as prior self-harm episodes, comorbid alcohol misuse and psychiatric treatment in South Asian and Black people compared with White people (Turnbull *et al*., 2015). Associations are, however, also likely to be complex. For example whereas cardiovascular disease is known to be more prevalent across ethnic minority populations (Yusuf *et al*., 2001) and especially so in ethnic minority groups with severe mental disorders (Das-Munshi *et al*., 2017*a*), lower levels of smoking and smoking-related disease (such as Chronic Obstructive Airways Disease, COPD) (Gilkes *et al*., 2017) have also been noted among ethnic minority group in the UK.

Several studies have also suggested potential beneficial effects for people living in areas of higher own ethnic density for lowering suicide risk (Neeleman and Wessely, 1999; Termorshuizen *et al*., 2015) and suicidal ideas (Bécares and Das-Munshi, 2013), as well as potentially even lowering the risk of detrimental health behaviours such as tobacco use (Mathur *et al*., 2017) and alcohol misuse (Bécares *et al*., 2011). The possibility that broader social factors alongside ethnic density, for example relating to social support, community-level cohesion and support networks, may also mitigate against other causes of death could be investigated and may help to shed light on the broader determinants of mortality in people with depression.

#### Variations in SMRs by ethnicity in depression

Cause-specific SMRs were similar across each of the ethnic minority groups, although of note the SMR for suicide was elevated in the Irish group with depression. The finding in the Irish group is consistent with previous research which has highlighted elevated mortality in Irish-born and second and third generation Irish people in the UK, including deaths from suicide (Harding and Balarajan, 2001; Maynard *et al*., 2012). The reasons for this across generations are complex and may include challenges related to creating an ‘authentic identity’(Leavey *et al*., 2004; Ryan *et al*., 2018), unplanned migration, especially amongst Irish-born men (Ryan *et al*., 2018), earlier childhood experiences of adversity in the second generation, related to the stressful parental experiences of migration and settlement in the Irish-born (Das-Munshi *et al*., 2013; Das-Munshi *et al*., 2014*a*), and health-related behaviours such as alcohol use which are associated with an increased risk of depression, and used as a means of coping with loneliness and other social hardships (Leavey *et al*., 2007) and itself associated with earlier childhood experiences (Das-Munshi *et al*., 2014*b*). Far less work has been conducted on Chinese people in Britain, the sample size in the present study limited inferences, but there was a suggestion of elevated suicide SMRs in this group, and this should be explored in future research. Conversely, deaths from respiratory disorders appeared slightly lower in the Black Caribbean and Black African groups with depression. These findings reflect previous work which has highlighted lower rates of tobacco use in Black African people and a lower prevalence of Chronic Obstructive Airways diseases in Black African and Black Caribbean groups (Gilkes *et al*., 2017; Mathur *et al*., 2017). The finding of a lower cancer SMR in the South Asian group with depression is consistent with the literature which has suggested lower cancer mortality in South Asian-born populations relative to local-born populations in the UK and other European countries (Ikram *et al*., 2016).

#### Other associations with mortality in depression

Other findings also broadly confirm the wider literature: male sex was associated with an increased all-cause, natural cause and unnatural cause mortality risk (Oquendo *et al*., 2001; Cuijpers and Smit, 2002; Mykletun *et al*., 2009; Pratt *et al*., 2016), with near-double elevated risks for all-cause and unnatural causes; alcohol/substance misuse was associated with an increased risk of death for all-cause and unnatural-causes (Zivin *et al*., 2007; Hjorthøj *et al*., 2015). Residence in less deprived areas was associated with a reduced risk of death from all-cause and natural-cause mortality. The finding in our study of a reduced mortality risk in depression with comorbid anxiety, relative to remaining cases of depression, is consistent with those of a previous population-based study in which the investigators found protective associations for mortality for depression comorbid with case-level anxiety (Mykletun *et al*., 2009). Investigators of a more recent study of secondary care depression also did not find that the presence of anxiety comorbidities increased the risk of mortality in secondary care depression, rather the presence of comorbid anxiety was associated with a reduction in mortality risk in depressed subjects from respiratory disorders and suicide (Laan *et al*., 2011). However, it is also worth noting that in one other study the investigators reported that the presence of comorbid anxiety with depression increased mortality risk, relative to the general population (Meier *et al*., 2016). In this study, the authors utilised nationwide data from Denmark and restricted the population to under 57 years of age. The differences between our studies may be due to the use of a different reference group (the investigators compared comorbid depression/anxiety to population controls, whereas we utilised internal comparisons in a cohort with depression) and a focus on younger adults.

### Strengths and limitations

The relatively large size of the cohort of 20 320 individuals with unipolar depression, with 2366 deaths over the 8-year follow-up period meant that the study was, in general, adequately powered to assess differences in mortality experience for each of the covariates with all-cause mortality outcomes, although for some groups (e.g. Chinese and Irish people) smaller sample sizes limited inferences. The cohort was from an ethnically diverse urban area in the UK, and as such would have good generalisability to other such metropolitan locations. In addition, the linkage with NHS identifiers meant that we were able to adjust for the competing risk of emigration out of the cohort, and so were able to adjust for the potential impact of return migration bias (or ‘salmon effect bias’) (Razum, 2006), but despite these, adjustments did not find that estimates were impacted. The prospective design permitted an assessment of the impact of time on mortality outcomes in depression, indicating that the increased all-cause mortality risk in men compared with women, reduced over time. The validation study for depression confirmed high sensitivity and specificity for this diagnosis within the records.

The assessment of associations for the rarer outcome of unnatural-causes mortality was affected by low power, which impacted adversely on precision. Additional limitations include the possibility of confounding by variables we did not have detail on, as the study was based on electronic health records. Work is currently underway on the data resource to use NLP for more detailed phenotypic and comorbidity profiling, so it may be possible in future to assess potential modifying and mediating factors in more detail (Perera *et al*., 2016). Future data linkages may enable a more detailed assessment of individual-level social indicators for mortality risks in depression; in these analyses, we were restricted to utilising area-level indices of multiple deprivation. It is a limitation that we currently do not have consistently captured data on physical comorbidities which are known to play a major role in the increased risk of mortality in unipolar depression. It may be possible to assess physical health comorbidities as mediating associations in future work on this cohort, as work is currently underway to develop NLP applications to extract this information from free text (Perera *et al*., 2016). Also as we only identified comorbid depression/anxiety cases if occurring either at the same time as, or after the index depression diagnosis, it is possible that we under-estimated the presence of comorbid anxiety. Future work could explore the timing of anxiety/depression comorbid diagnoses, and their relationship to adverse outcomes such as mortality.

Potential sources of bias should also be considered, especially those relating to the lower observed mortality risk in ethnic minority groups relative to the White British group with depression. First, in the UK most cases of depression are managed in primary care. Our study focussed on secondary care, and as such, reflects people who would have had complex and/or moderate to severe depression, referred by primary care into secondary care. Although the healthcare provider provides near-monopoly mental health care coverage to the geographical area, it is also possible that some people with depression may have sought private healthcare, and we would not have had detail on these individuals. The healthcare system in the UK is free at the point of contact, so this would have minimised the risk of this possibility. It is possible that ethnic minority groups with depression may have been less likely to have been referred/accessed secondary care, either through being less likely to health-seek or because of the ‘gate-keeper’ function of general practitioners/primary care. Investigators in a previous study, conducted in part of the catchment area for the present study, found that people with common mental disorders who self-referred for psychological therapies did not differ by mean age, gender or ethnicity, compared with a sample of people who screened positive for common mental disorders, from a representative community survey of individuals from the same catchment area (Brown *et al*., 2014). The investigators found, however, that individuals referred for psychological therapies via their general practitioner/family doctor (rather than through self-referral) did differ on these characteristics, in particular with a lower proportion of ethnic minority groups referred onwards. If the findings from this previous study are taken into account, we may assume low levels of differences in actual health seeking for common mental disorders by ethnicity and other characteristics in our sample, however the role of bias due to the gate-keeper function of general practitioners is a concern and is difficult to quantify, although it seems unlikely that ethnic minority groups with less severe forms of depression (hence associated with lower mortality risks (Mykletun *et al*., 2009)) would have been preferentially referred to secondary care. In addition, susceptible individuals may have selected themselves out of the cohort/population at risk prior to the depressive illness being notified on to the electronic health records system. As we were unable to account for prior events it is possible that our comparison of mortality risk across groups may have been confounded by this.

Finally, it is also possible that lower mortality risks in the ethnic minority groups relative to the White British group with depression were a function of differential pathways into care. If individuals from an ethnic minority background had higher levels of physical health comorbidities, they may have been more likely to have been in contact with their general practitioner/family doctor and/or general medical services, and therefore had physical health problems addressed in a more timely manner (leading to lower mortality risks) than White British people with depression. We plan to assess this possibility in future research.

## Conclusions

In conclusion, our findings suggest that depression may indeed be a ‘life threatening’ disorder (Cuijpers and Smit, 2002), however, with the caveat that there are important factors relating to ethnicity, sex, relationship status, comorbidities and residency in deprived neighbourhoods, which are associated with this increased risk and, as this was an observational study, causal inferences may still have been hampered by residual confounding, so results should be interpreted with caution. Future work should attempt to identify aetiological factors which may play a role in improving mortality outcomes in individuals with major depression.

## Supplementary material

For supplementary material accompanying this paper visit https://doi.org/10.1017/S0033291718002210. click here to view supplementary material

## Data access

Data are owned by a third party, Biomedical Research Centre (BRC) Clinical Records Interactive Search (CRIS) tool, which provides access to anonymised data derived from SLaM electronic medical records. The authors do not have access to de-identified individual-level data. Only approved researchers can access anonymised data for approved projects.
