# Supplementary material for: Depression and cause-specific mortality in an ethnically diverse cohort from the UK: 8-year prospective study
Source: Psychol Med. 2018 Sep 5;49(10):1639–51. doi: 10.1017/S0033291718002210 (PMC6601358; doi:10.1017/S0033291718002210)
Supplement: Supplementary file 1 [file S0033291718002210sup001.docx]

**Supplementary material**

**Table 1**

**Association of sex with all-cause mortality over time in unipolar depression (N=20,320)**

|  |  | **Time in study** | |  |  | |  |  | |  |
| --- | --- | --- | --- | --- | --- | --- | --- | --- | --- | --- |
|  |  | **0-1 years** | |  | **1-3 years** | |  | **3 years+** | |  |
|  |  | **HR** | **(95% CI)** | **p value** | **HR** | **(95% CI)** | **p value** | **HR** | **(95% CI)** | **p value** |
| Crude/ unadjusted | Female | 1.00 | REF | . | 1.00 | REF | . | 1.00 | REF | . |
|  | Male | 1.81 | (1.57, 2.08) | <0.0001 | 1.31 | (1.14, 1.51) | <0.0001 | 1.10 | (0.96, 1.27) | 0.18 |
|  | Female | 1.00 | REF | . | 1.00 | REF | . | 1.00 | REF | . |
| Adjusted^†^ | Male | 1.94 | (1.68 ,2.24) | <0.0001 | 1.53 | (1.32, 1.76) | <0.0001 | 1.34 | (1.16, 1.55) | <0.0001 |

***Key:*** *HR: Hazard Ratios; ^†^Adjusted for ethnicity, relationship status, comorbid anxiety, comorbid alcohol or substance misuse, area-level deprivation and age; Likelihood Ratio Test (LRT) for p value for interaction of sex*time: <0.001*

**Supplementary material**

**Table 2**

**All-cause mortality associations in unipolar depression;**

**Sensitivity analyses** *^†^*

|  |  | **Deaths** | **sHR** | **(95% CI)** | **p value** |
| --- | --- | --- | --- | --- | --- |
| Ethnicity | White British | 14.2% | REF |  |  |
|  | Irish | 19.2% | 0.87 | 0.74 ,1.03 | 0.10 |
|  | Black Caribbean | 5.2% | 0.62 | 0.53 ,0.73 | <0.0001 |
|  | Black African | 2.4% | 0.52 | 0.39 ,0.71 | <0.0001 |
|  | South Asian | 7.2% | 0.69 | 0.52 ,0.90 | 0.006 |
|  | Chinese | 4.3% | 0.65 | 0.27 ,1.58 | 0.34 |
| Sex^‡^ | Female | 10.5% | REF |  |  |
|  | Male | 13.7% | 1.95 | 1.68 ,2.26 | <0.0001 |
| Relationship status | Married/ cohabiting | 15.3% | REF |  |  |
|  | Divorced/ separated | 10.2% | 0.98 | 0.84 ,1.15 | 0.83 |
|  | Single | 5.5% | 1.16 | 1.03 ,1.32 | 0.017 |
|  | Widowed | 48.5% | 1.35 | 1.20 ,1.52 | <0.0001 |
|  | Civil partnership | 11.0% | 1.31 | 1.11 ,1.54 | 0.001 |
| Comorbid anxiety | No comorbid anxiety | 12.0% | REF |  |  |
|  | Comorbid anxiety | 6.9% | 0.72 | 0.57 ,0.90 | 0.004 |
| Substance misuse | No alcohol/ substance misuse | 12.3% | REF |  |  |
|  | Lifetime alcohol/illicit substance misuse | 8.0% | 1.18 | 1.01 ,1.36 | 0.032 |
| Area level deprivation | Most deprived fifth | 11.9% | 0.96*^¥^* | 0.93 ,0.99 | 0.004 |
|  |  | 11.0% |  |  |  |
|  |  | 10.5% |  |  |  |
|  |  | 11.0% |  |  |  |
|  | Least deprived fifth | 14.0% |  |  |  |

***Key:*** *sHR: Hazard Ratios; ^†^Competing risk models adjusted for ethnicity, relationship status, comorbid anxiety, comorbid alcohol or substance misuse, area-level deprivation, age with sex by time interaction, sub-Hazard Ratios take into account competing risk of emigration out of the cohort; ^¥^ Linear trend association with mortality, per increase in area-level deprivation (fifths); ^‡^Displayed sHRs in table for sex were at 0-1 years in study, sHRs for (men versus women) at 1-3 years were 1.52 (95% CI: 1.32, 1.75 and at 3 years+ sHR: 1.37 (95% CI: 1.18, 1.58)*

**Supplementary material**

**Table 3**

**Standardised Mortality Ratios for all-cause mortality in unipolar depression (N=20,320)***

|  | **Observed/ expected deaths** | **Standardised to the population of England & Wales** | **Standardised**  **to local area†** |
| --- | --- | --- | --- |
| Total sample | 2366/ 929 | 2.55 (2.45 2.65) | 2.65 (2.54, 2.76) |
| White British | 1966/ 747 | 2.63 (2.52 2.75) | 2.74 (2.62, 2.86) |
| Black Caribbean | 153/ 72 | 2.13 (1.81 2.50) | 2.18 (1.85, 2.56) |
| Black African | 45/ 21 | 2.19 (1.60 2.94) | 2.26 (1.65, 3.03) |
| South Asian | 53/ 24 | 2.17 (1.62 2.83) | 2.24 (1.68, 2.93) |
| Irish | 144/ 63 | 2.28 (1.93 2.69) | 2.40 (2.03, 2.83) |
| Chinese | ≤10* | 2.11 (0.69 4.93) | 2.16 (0.70, 5.04) |

**Key**

**Age (ten year bands)- and sex- standardised*

*†Population of the London boroughs of Lambeth, Lewisham, Southwark and Croydon*

**Supplementary material**

**Table 4**

**Performance of Natural Language Process (NLP) algorithm for depression diagnoses against clinician gold standard in the sample, by ethnic**

**group**

|  | **Full sample** | **White British** | **Irish** | **Black Caribbean** | **Black African** | **South Asian** | **Chinese** |
| --- | --- | --- | --- | --- | --- | --- | --- |
| **Sensitivity** | 89.6%  (95% CI: 77.3%, 96.5%) | 80.0%  (95% CI: 59.3, 93.2) | 91.7%  (95% CI: 61.5, 99.8) | 94.1%  (95% CI:71.3,99.6) | 89.0%  (95% CI: 65.2, 98.6) | 100%  (95% CI:69.2, 100) | 94.4%  (95% CI: 72.7, 99.9) |
| **Specificity** | 88.0%  (95% CI: 75.6%, 95.5%) | 87.1%  (95% CI: 70.2, 96.4) | 88.9%  (95% CI: 65.3, 98.6) | 86.4%  (95% CI:65.1,97.1) | 78.6%  (95% CI: 49.2, 95.3) | 93.8%  (95%CI: 69.8, 99.8) | 81.8%  (95% CI: 59.7, 94.8) |
| **Positive predictive value** | 87.8%  (95% CI: 75.2%, 95.4%) | 83.3%  (95% CI: 62.6, 95.3) | 84.6%  (95% CI:54.6, 98.1) | 84.2%  (95% CI:60.4,96.6) | 84.2%  (95% CI: 60.4, 96.7) | 90.9%  (95%CI: 58.7, 99.8) | 81.0%  (95%CI: 58.1, 94.6) |
